# Cross Kingdom Activators of Five Classes of Bacterial Effectors

**DOI:** 10.1371/journal.ppat.1004944

**Published:** 2015-07-23

**Authors:** David M. Anderson, Jimmy B. Feix, Dara W. Frank

**Affiliations:** 1 Department of Microbiology and Molecular Genetics, Medical College of Wisconsin, Milwaukee, Wisconsin, United States of America; 2 Center for Infectious Disease Research, Medical College of Wisconsin, Milwaukee, Wisconsin, United States of America; 3 Department of Biophysics, Medical College of Wisconsin, Milwaukee, Wisconsin, United States of America; The University of North Carolina at Chapel Hill, UNITED STATES

## Introduction

**Fig 1:**
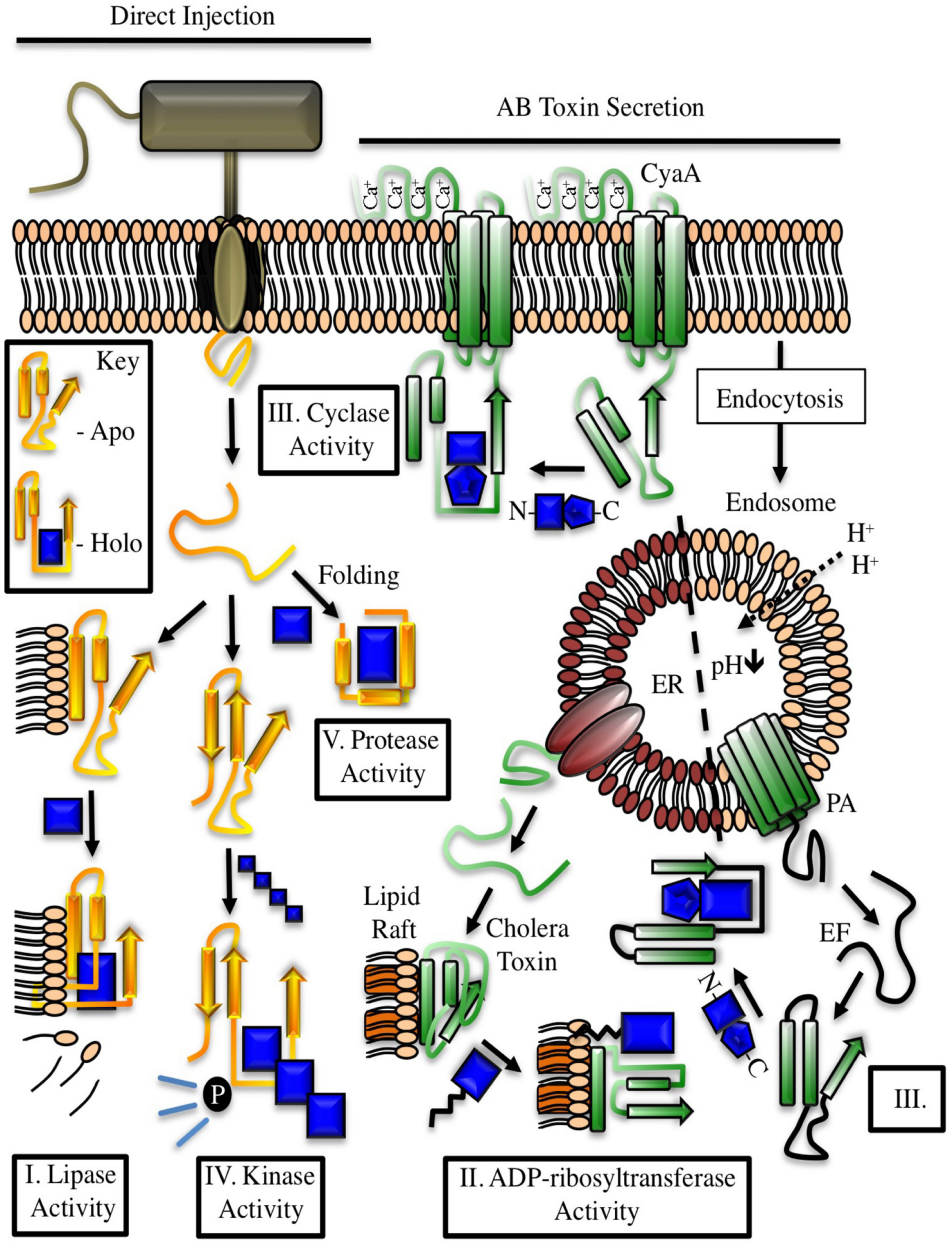
Examples of cofactor regulation of secreted bacterial enzymes. Toxins delivered to their target cell by either direct injection (yellow) or cell surface binding and translocation (green) can be host cofactor activated. These enzymes generally contain dynamic structures that assume a catalytically active fold upon complex formation with a host factor. The key depicts this process through cofactor-mediated organization (blue) of an unstructured sequence. Apo, the apoenzyme catalytically-inactive state. Holo, the holoenzyme active state in which the toxin is in complex with its cofactor. CyaA, the plasma membrane-localized nucleotide cyclase toxin from *Bordetella pertussis* complexed to calcium ions and calmodulin. EF, edema factor from *Bacillus anthracis*, binds to calmodulin in a different orientation than CyaA. PA, protective antigen. The cofactor for cholera toxin (ARF) is shown in the myristolated, GTP-bound form. ER, endoplasmic reticulum, with cholera toxin peptide being secreted through ER protein channels.

Toxin production by pathogenic microorganisms likely serves to (i) protect against phagocytosis by predatory cells, (ii) aid in penetrating tissue barriers, (iii) promote nutrient release, or (iv) alter cellular architecture and metabolism in ways that facilitate the establishment of a niche for colonization and replication. Useful enzymatic targets for some bacterial toxins may be similar between prokaryotic and eukaryotic environments, necessitating a need for eukaryotic-specific cofactors to regulate toxin activity. Host cell—derived factors may impart localization properties to an effector, induce folding events, provide a platform for the inhibition of cellular processes or support greater substrate promiscuity (Fig 1). The aim of this review is to describe the diversity of bacterial effectors known to require, or to be stimulated by eukaryotic cofactors and to integrate new ideas regarding the structural and functional implications of this relationship (Table 1).

**Table 1:** Cross kingdom enzyme stimulators for secreted bacterial toxins.

Strain	Enzyme	Stimulated Activity	Activator
*Pseudomonas aeruginosa*	ExoU	PLA2/Lyso-PLA	Ubiquitin
*Pseudomonas fluorescens*	ExoU-like	PLA2	Ubiquitin
*Photorhabdus asymbiotica*	LopU	PLA2	Ubiquitin
*Burkholderia thailandensis*	ExoU-like	PLA2	Ubiquitin
*Rickettsia prowazekii*	RP534	PLA1/PLA2/Lyso-PLA2	bSOD(ubiquitin)
*Rickettsia typhi*	RT0590/Pat1	PLA2	bSOD(ubiquitin)
*Rickettsia typhi*	RT0522/Pat2	PLA2	bSOD(ubiquitin)
*Legionella pneumophila*	VipD	PLA1	Rab5
*Salmonella typhimurium*	SseJ	Esterase	GTP-RhoA
*Bordetella pertussis*	CyaA	Nucleotidyl cyclase	Calmodulin
*Bacillus anthracis*	Edema factor	Nucleotidyl cyclase	Calmodulin
*Pseudomonas aeruginosa*	ExoY	Nucleotidyl cyclase	Actin?
*Aeromonas hydrophila*	AexU	ADP-ribosyltransferase	14-3-3
*Aeromonas* spp.	AexT	ADP-ribosyltransferase	14-3-3
*Pseudomonas aeruginosa*	ExoS	ADP-ribosyltransferase	14-3-3
*Pseudomonas aeruginosa*	ExoT	ADP-ribosyltransferase	14-3-3
*Vibrio parahaemolyticus*	VopT	ADP-ribosyltransferase	14-3-3
*Vibrio cholerae*	Cholera toxin	ADP-ribosyltransferase	ARF
*Escherichia coli*	Heat labile toxin	ADP-ribosyltransferase	ARF
*Shigella flexneri*	OspG	Kinase	E2-Ubiquitin
*Yersinia pestis*	YopO/YpkA	Kinase	G-actin
*Pseudomonas syringae*	HopZ1	Acetyl transferase	IP6
*Yersinia* spp.	YopJ	Acetyl transferase	IP6
*Salmonella typhimurium*	AvrA	Acetyl transferase	IP6
*Vibrio parahaemolyticus*	VPA1380	Cysteine protease	IP6
*Pseudomonas syringae*	AvrRpt2	Cysteine protease	Cyclophilin
Totals	26	9	12

### I. Phospholipases

Many bacterial toxins contain catalytic domains with homology to plant patatins, which are lipid acyl hydrolases found in potato tubers. Cofactor activation of phospholipase activity is best characterized for ExoU, a lipid membrane—hydrolyzing protein encoded by the opportunistic pathogen *P*. *aeruginosa*. Monoubiquitin, ubiquitin polymers, and ubiquitylated proteins are capable of activating ExoU [1]. Bioinformatic analyses identified at least 17 additional bacterial patatin-like phospholipases that fit the criteria for ubiquitin-mediated activation [2]. Functional studies of a selected subset of enzymes demonstrated that ubiquitin activates phospholipases from *P*. *asymbiotica*, *B*. *thailandensis*, and *P*. *fluorescens* [2].

ExoU orthologs are also found in frank pathogens within the Rickettsiae, Legionellae, and Salmonellae. *R*. *typhus* encodes ExoU homologs, Pat1 and Pat2 [3,4]. Both proteins are activated by preparations of bovine SOD1 (bSOD1 [3,4]), which may provide a source of ubiquitin [1]. Additionally, *R*. *prowazekii*, the etiologic agent of typhus, displays enhanced enzymatic activity in the presence of bSOD1 (ubiquitin, [5]). *Rickettsial*-derived PLA enzymes function in cellular entry [6], phagosomal escape, and noncytolytic free fatty acid release [7].

VipD lipase from *L*. *pneumophila* displays a Rab5-dependent PLA1 activity [8] that targets the enzyme to endosomes resulting in inhibition of phagosome maturation. SseJ, a glycerophospholipid-cholesterol acyltransfersase (GCAT)-like enzyme from *Salmonella*, is activated by GTP-RhoA [9] likely for the temporal regulation of cholesterol metabolism in infected cells [10]. The *Y*. *enterocolitica* homolog to SseJ, YspM, may have a similar function [11].

### II. Transferases

Most cofactor-stimulated transferases catalyze the transfer of an ADP-ribose moiety from nicotinamide adenine dinucleotide to a target protein. Exoenzyme S from *P*. *aeruginosa* was one of the first identified bacterial ADP-ribosyltransferases requiring a cofactor for activation [12]. Members of the 14-3-3 family of eukaryotic scaffolding proteins stimulate ADP-ribosylation of a variety of targets causing disruptions in cytoskeletal integrity and vesicular trafficking [13]. Homologous enzymes are present in *A*. *hydrophila* [14] and *V*. *parahaemolyticus* [15].

Another enzyme from *P*. *aeruginosa*, ExoT, is similar (76% identity) to ExoS and requires 14-3-3 proteins as cofactors, but has limited target recognition and is not overtly cytotoxic [16]. CrkI and CrkII are the only proteins modified by ExoT [17]. ADP-ribosylation uncouples integrin signaling and alters the cytoskeleton. *A*. *hydrophila* and *A*. *salmonicida* encode enzymes similar to ExoT/S termed AexT [18].

Bacterial effectors with acetyltransferase activity include YopJ, a type III secreted enzyme from *Yersinia* that inhibits immune responses through acetylation of several residues in the kinases involved in the MAPK and NFκB pathways [19]. Acetyltransferase activity is stimulated by a highly abundant eukaryotic signaling carbohydrate, inositol hexakisphosphate (IP6, [20]). *P*. *syringae* secretes an IP6-activated acetyltransferase, HopZ1, which interferes with plant cell cytoskeletal networks and downstream cell wall—mediated immune responses by acetylating microtubules [21]. The binding of IP6 to *S*. *typhimurium* AvrA suggests that stimulation of acetyltransferase activity in this family of enzymes likely involves allosteric conformational changes [20]. Structural investigations of *V*. *cholerae* RTX toxin [22] and *Clostridium difficile* toxin A [23] suggest that IP6 interactions with bacterial enzymes, and their subsequent allosteric conformational changes, may be a conserved strategy.

ADP-ribosylation factors (ARFs) have been identified as stimulators of type I and type II heat labile toxin (LT) activity from enteric bacteria. Cholera toxin from *V*. *cholerae*, LT-I and LT-II from *E*. *coli*, are the most studied enzymes of this family to date; each are highly homologous in sequence and structure. Cholera toxin A1 subunit was shown to fold on host cell lipid rafts [24] before interacting with ARF proteins in their GTP-bound state [25]. Activated enzymes ADP-ribosylate the alpha subunit of the heterotrimeric G stimulatory protein (G_s_), locking it into the GTP-bound form. This stimulates adenylyl cyclase activity and generates supraphysiological amounts of cAMP, deregulating the chloride balance in the intestinal lumen to cause diarrhea.

### III. Nucleotide Cyclases

Cofactor-stimulated nucleotide cyclases include the adenylyl cyclases edema factor (EF) of anthrax toxin and CyaA of *B*. *pertussis*. These enzymes contain structurally similar components but differ in affinity and binding mechanism for their eukaryotic activator, calmodulin (CaM). Edema factor has three domains: a protective antigen-binding domain, the adenylyl cyclase core domain, and an alpha-helical domain. The helical domain interacts with the core domain to maintain an unstructured activation loop. The N-terminal domain of CaM interacts with the EF helical domain, causing a conformational switch between the helical and core domains [26]. The C-terminal domain of CaM then docks into a crevice between the helical and core domains, stabilizing the catalytic loop [27].

In contrast to soluble EF, CyaA localizes to the cytosolic interface at the plasma membrane. The CaM binding domain involves completely different sequences with an affinity that is roughly 100-fold stronger as compared to EF. N- and C-terminal domains of CaM participate in binding, stabilization, and activation of CyaA. The C-terminal domain stabilizes a catalytic loop, and the N-terminal domain is postulated to bind a β-hairpin motif of CyaA to promote substrate (ATP) binding within the catalytic chamber [28].

Exoenzyme Y is a type III-secreted, cofactor-stimulated adenylyl cyclase from *P*. *aeruginosa* [29]. Functionally, ExoY cyclase activity disrupts the actin cytoskeleton and is correlated with Tau hyperphosphorylation, which leads to microtubule breakdown, endothelial cell gap formation, and increased tissue permeability [30]. The cofactor for ExoY remains to be established, but preliminary work suggests that host cell actin stimulates cyclase activity [31]. Sensitive detection methods demonstrate that ExoY, EF, and CyaA have broad nucleotidyl cyclase activity and catalyze the formation of cCMP and cUMP (or cGMP and cUMP with ExoY) in addition to cAMP [32,33]. The production of cCMP and cUMP during in vivo infection may provide an experimental system to explore the roles of these cyclic nucleotides as new second messengers [34].

### IV. Kinases

*Yersinia* species inject actin-stimulated effector kinases into host cells [35]. YopO or YpkA binds to actin in a manner that does not interfere with the association of other actin-targeting proteins, while simultaneously blocking actin incorporation into a filament. Thus, actin facilitates catalysis by stabilizing the enzyme’s catalytic loop while serving as a platform for phosphorylation of other actin-binding proteins [36].

Another kinase characterized from *Shigella* species, but also present in strains of *Yersinia* and enterohemorrhagic *E*. *coli*, is OspG [37–39]. OspG is involved in attenuating NFκB signaling by interfering with IκBα degradation [40]. Ubiquitin and ubiquitin chains were first identified as stimulators of OspG kinase activity in vitro [39]. Two groups subsequently solved structures of OspG in complex with E2 ubiquitin-conjugating enzymes covalently linked to monoubiquitin, UbcH5a~Ub [38] and UbcH7~Ub [37]. At least 10 different E2~Ub conjugates appear to be able to activate OspG, suggesting that there may not be a strict preference towards a particular E2.

### V. Proteases

*V*. *parahaemolyticus* has recently been shown to secrete an IP6-activated enzyme, VPA1380, with homology to the cysteine protease domains of multifunctional-autoprocessing RTX (MARTX) domains from *Vibrio* and large clostridial toxins A and B [41]. The mechanism of activation, toxicity, and target substrates are yet to be elucidated. *P*. *syringae* secretes a protease, AvrRpt2, which is homologous to the staphopain cysteine proteases that are activated through interactions with cyclophilins [42]. AvrRpt2 is predominantly in an unstructured coil conformation until binding to a host cyclophilin, which induces folding into a stable conformer [43]. Analysis of protease activity supports a model in which AvrRpt2 must be in complex with cyclophilin cofactor for maximal enzymatic activity [44].

## Conclusions

Numerous secreted bacterial enzymes interact with host cell factors to ensure enzymatic activation in the correct environment, and in some cases, trafficking to a specific location within the host. Common properties of the activators are that they are ubiquitously distributed throughout the eukaryotic kingdom, are present in high concentration, and play roles in cell signaling. The prevailing theme of a high cellular concentration may facilitate the required conditions for toxins to establish a cross kingdom binding partner as they evolved [45]. In this way, highly regulated and flexible proteins that are easily translocated across lipid barriers could be allosterically reorganized to a stable and active conformation. Importantly, bacterial utilization of host cell machinery to ensure maximal toxin activity in the correct environment minimizes the maintenance of bacterial genetic information.
